# The Effect of Complex Interventions on Depression and Anxiety in Chronic Obstructive Pulmonary Disease: Systematic Review and Meta-Analysis

**DOI:** 10.1371/journal.pone.0060532

**Published:** 2013-04-05

**Authors:** Peter A. Coventry, Peter Bower, Christopher Keyworth, Cassandra Kenning, Jasmin Knopp, Charlotte Garrett, Daniel Hind, Alice Malpass, Chris Dickens

**Affiliations:** 1 Collaboration for Leadership in Applied Health Research and Care for Greater Manchester, Centre for Primary Care, and Manchester Academic Health Science Centre, University of Manchester, Manchester, United Kingdom; 2 National Institute for Health Research School for Primary Care Research, Centre for Primary Care, and Manchester Academic Health Science Centre, University of Manchester, Manchester, United Kingdom; 3 Institute of Inflammation and Repair and Manchester Academic Health Science Centre, University of Manchester, Manchester, United Kingdom; 4 Institute of Population Health, Centre for Primary Care, and Manchester Academic Health Science Centre, University of Manchester, Manchester, United Kingdom; 5 Clinical Trials Research Unit, School of Health and Related Research, University of Sheffield, Sheffield, United Kingdom; 6 School of Social and Community Based Medicine, University of Bristol, Bristol, United Kingdom; 7 Mental Health Research Group, Peninsula College of Medicine and Dentistry, and National Institute for Health Research Collaboration for Leadership in Applied Health Research and Care for the South West, University of Exeter, Exeter, United Kingdom; Baylor College of Medicine, United States of America

## Abstract

**Background:**

Depression and anxiety are very common in people with chronic obstructive pulmonary disease (COPD) and are associated with excess morbidity and mortality. Patients prefer non-drug treatments and clinical guidelines promote non-pharmacological interventions as first line therapy for depression and anxiety in people with long term conditions. However the comparative effectiveness of psychological and lifestyle interventions among COPD patients is not known. We assessed whether complex psychological and/or lifestyle interventions are effective in reducing symptoms of anxiety and depression in patients with COPD. We then determined what types of psychological and lifestyle interventions are most effective.

**Methods and Findings:**

Systematic review of randomised controlled trials of psychological and/or lifestyle interventions for adults with COPD that measured symptoms of depression and/or anxiety. CENTRAL, Medline, Embase, PsychINFO, CINAHL, ISI Web of Science and Scopus were searched up to April 2012. Meta-analyses using random effects models were undertaken to estimate the average effect of interventions on depression and anxiety. Thirty independent comparisons from 29 randomised controlled trials (n = 2063) were included in the meta-analysis. Overall, psychological and/or lifestyle interventions were associated with small reductions in symptoms of depression (standardised mean difference −0.28, 95% confidence interval −0.41 to −0.14) and anxiety (standardised mean difference −0.23, 95% confidence interval −0.38 to −0.09). Multi-component exercise training was the only intervention subgroup associated with significant treatment effects for depression (standardised mean difference −0.47, 95% confidence interval −0.66 to −0.28), and for anxiety (standardised mean difference −0.45, 95% confidence interval −0.71 to −0.18).

**Conclusions:**

Complex psychological and/or lifestyle interventions that include an exercise component significantly improve symptoms of depression and anxiety in people with COPD. Furthermore, multi-component exercise training effectively reduces symptoms of anxiety and depression in all people with COPD regardless of severity of depression or anxiety, highlighting the importance of promoting physical activity in this population.

## Introduction

One in four patients with chronic obstructive pulmonary disease (COPD) will have clinically significant depression, which is twice the prevalence observed in people without COPD [1]. Similarly, compared with matched controls, people with COPD are at least twice as likely to experience anxiety [2]. Inflammatory and physiologic changes associated with COPD have been implicated in the onset of depression and anxiety [3], although there is strong evidence to suggest that subjective health status is a better predictor of depression in COPD than biological or physiological markers [4].

Irrespective of cause, depression and anxiety have profound consequences for the health of patients with COPD. Depression is associated with increased mortality, impaired health related quality of life and longer hospital stay after acute exacerbation [5], increased risk of exacerbation and hospital admission [6], hospital readmission [7], and poorer exercise performance [8]. Equally, anxiety is associated with increased risk of exacerbations, poorer health related quality of life and worse exercise performance [2], relapse within one-month of receiving emergency treatment [9], and hospital readmission [10].

In the UK, the National Institute for Health and Clinical Excellence have published guidelines for treating depression and anxiety in people with long term conditions [11]. Treatments include psychological therapies with or without antidepressant medication. Importantly the National Institute for Health and Clinical Excellence guideline for COPD emphasises offering patients psychological and psychosocial interventions, including behavioural approaches such as pulmonary rehabilitation, before considering antidepressants [12].

However, the comparative effects of different psychological interventions remains uncertain in long term conditions, and the relevance of systematic review data is largely confined to treatment of depression rather than both depression and anxiety, which commonly coexist in people with long term conditions [11]. Moreover, the evidence for using psychological interventions in COPD patients is equivocal. While there is some support for treating depression and anxiety in COPD using cognitive and behavioural therapy (CBT), (with or without exercise or education), evidence is largely derived from either small randomised controlled trials or uncontrolled and non-randomised studies [13], [14]. Whereas Rose et al found insufficient evidence to support the use of psychologically-based treatments to reduce anxiety [15], a more recent meta-analysis of eight psychotherapeutic and one relaxation intervention reported a small but significant effect on anxiety (*r* = −0.27, 95% confidence interval −0.41 to −0.14), but not depression [16]. Previous reviews of pulmonary rehabilitation have indicated that 4-week programmes can improve fatigue and emotional function, but these reviews either included trials that did not specifically address effects on anxiety and depression [17], or included non-randomised trials known to be affected by selection bias [18].

Attempts to systematically review and quantify the effectiveness of a more broad range of complex, non-pharmacological interventions, including psycho-educational and lifestyle interventions, on mental health in COPD have similarly been confounded by methodologically heterogeneous approaches [19], [20], leading to uncertainty about which interventions to use in this population. We have therefore conducted a systematic review and meta-analysis of randomised controlled trials of complex psychological and/or lifestyle interventions for managing COPD. Our first objective was to assess whether complex interventions that incorporate psychological and/or lifestyle components are effective in reducing symptoms of anxiety and depression in patients with COPD. Secondly, we determined what types of complex psychological and/or lifestyle interventions are most effective.

## Methods

This systematic review is reported in accordance with the Preferred Reporting Items for Systematic Reviews and Meta-Analyses Statement (Appendix S1) [21]. No formal protocol was published.

### Information Sources and Search Strategy

The following electronic databases were searched: Cochrane Central Register of Controlled Trials (CENTRAL, issue 10, 2010), Medline In-Process and Other Non-Indexed Citations (Ovid) and Medline (Ovid) from inception to January 2011, Embase (Ovid) from inception to January 2011, PyschINFO (Ovid) from inception to January 2011, Cinahl (Ovid), 1981 to January 2011, ISI Web of Science 1945 to January 2011, and Scopus. In addition we searched reference lists of included studies and of three reviews of psychological management of COPD that were not identified in the electronic search [22]–[24]. All searches were carried out between October 2010 and January 2011 and updated in April 2012. Non-English publications were translated. The full search strategy for each database is available in Appendix S2.

### Eligibility Criteria

Studies were eligible for inclusion in this review if they met the following criteria:

Study design: cluster or individual randomised controlled trials.

Population: individuals with chronic obstructive pulmonary disease confirmed by post-bronchodilator spirometry of forced expiratory volume in 1 second/forced vial capacity ratio of <70% and a forced expiratory volume in 1 second of <80%.

Intervention: single or multiple component interventions that include psychological and/or lifestyle components to change knowledge, attitudes, beliefs, emotions, skills and/or behaviour in people with COPD. Studies that included patients treated for depression and/or anxiety with psychotropic medications were excluded. Interventions were classified based on an updated taxonomy of behaviour change techniques [25] (Table 1).

**Table 1:** Classification of intervention components.

Type of intervention	Description	Components
**Lifestyle**	General education	Basic provision of information, commonly using didactic techniques
	General discussion	Discussions facilitated by a professional or lay leader
	Exercise training	Illness-specific exercise
	Skills and self-management training	Teaching of practical skills to improve illness
	Behaviour therapy	Use of behavioural techniques, such as goal setting, to improve illness
	Relapse prevention	Discussion of how to maintain positive change and prevent future relapse
**Psychological**	Problem-solving techniques	Identification of problems/barriers to behaviour change and techniques to overcome them
	Cognitive behavioural therapy	Use or teaching of cognitive and behavioural techniques to invoke positive psychological change
	Social support	Use of teaching of techniques to improve social support
	Relaxation	Practice of relaxation techniques, including imagery and distraction
	Biofeedback	Biological feedback to support relaxation
	Miscellaneous	Mental health interventions lacking detailed description e.g. stress management

Comparators: any control (e.g. waiting list, usual care, attention or active control).

Outcomes: standardised measure of depression and/or anxiety.

We excluded studies and reports not published in peer reviewed journals, editorials, opinions and commentaries.

### Study Selection

Titles and abstracts were independently screened by four reviewers, and full papers of potentially relevant abstracts were retrieved. Full text versions of abstracts were independently screened and final decisions about eligibility were made at a consensus meeting with all review authors. Research staff with relevant language skills translated and interpreted non-English publications.

### Data Extraction

Data were extracted and cross-checked by pairs of reviewers using a standardised data extraction form used in a similar review of psychological interventions in diabetes [26]. Disagreements were resolved by discussion with two other reviewers (PC and CD). We contacted study authors to retrieve data not available in published study reports. Data were extracted on patient characteristics including age, gender, severity of COPD (classified according to the Global Initiative for Chronic Obstructive Lung Disease staging) [27], depression and anxiety severity at baseline, and whether patients were recruited on the basis of identified depression and/or anxiety. Data extracted on interventions included intensity (duration, number, and length of sessions), setting (e.g. primary care, community centre), mode of delivery (e.g. individual or group, face to face or remote delivery), and the professionals involved (e.g. mental health professional, respiratory nurse). Data on outcomes relevant to the review (post treatment depression and anxiety symptoms), comparator, and risk of bias were also extracted. Where studies combined COPD data with data for other respiratory disorders we wrote to authors to obtain separate data relevant to COPD patients.

### Risk of Bias

Risk of bias assessments were conducted independently for all included studies by two reviewers (CG and JK) using the Cochrane Collaboration tool [28]; discrepancies were resolved by discussion with a third reviewer (PC). Specifically, assessments were made that relate to: randomisation sequence generation, allocation bias, blinding of outcome assessment, losses to follow-up>20% [29], incomplete outcome data (adequate statistical handling of missing data), and intention-to-treat. Evaluations of risk of bias associated with blinding of outcome assessments and incomplete outcome data were restricted to depression and anxiety outcomes.

### Data Analysis and Synthesis

For each study that included continuous outcomes for depression and anxiety, a standardised mean difference (SMD) was calculated by taking the mean of the intervention group minus the mean of the control group, divided by the pooled standard deviation (SD). If there were several follow-ups we used the outcome data closest to post-treatment. Effect sizes expressed as SMDs are a useful method to compare the effect of an intervention across studies when different measures (such as different depression scales) are used. In keeping with established cut-offs of effect in behavioural medicine, effect sizes of 0.56 to 1.2 were categorised as large; effect sizes of 0.33 to 0.55 as moderate, and effect sizes ≤0.32 as small [30]. In this review, negative effect sizes indicated that the intervention improved depression and anxiety; statements about significance refer to statistical significance within 95% confidence intervals. Where exact means and SDs were missing from published reports or not provided by the authors (*k = *4) we estimated effect sizes using conventional methods [31], from exact *P* values [32], [33], and from a figure shown in the articles reviewed [34], [35]. If an SD was missing (*k* = 2) [36], [37] we imputed SDs from a comparable study in the meta-analysis that used the same measure [38]. Where trials reported two intervention groups and a single control group, separate SMDs were calculated for each intervention group but in the pooled analyses the sample size of the control group was halved to avoid double counting.

Meta-analyses using random effects models were undertaken to estimate the average effect of interventions on symptoms of depression and anxiety. Heterogeneity was analysed with the I^2^ index which represents the percentage of the total variability in a set of effect sizes due to between-study variability, rather than sampling error alone [39]; and by using Cochran’s *Q* test, which is calculated as the weighted sum of squared differences between individual study effects and the pooled effect across studies. The *Q* statistic follows a χ^2^ distribution with *k*-1 degrees of freedom, where *k* is equal to the number of studies contributing to the meta-analysis [31]; *Q*>*k* -1 suggests statistical heterogeneity with a cut-off value of 0.10.

The possibility of small publication bias (owing to the chance that significant studies are selectively published and not representative of all completed studies) was examined visually by scrutinising funnel plots and statistically using Egger’s test [40].

Meta-analysis and tests for small study bias were performed using Stata version 12 (Stata Corp. College Station, TX).

### Sensitivity and Subgroup Analysis

We carried out a pre-specified sensitivity analysis by removing studies where allocation concealment was either inadequate or not known from the overall pooled analysis to evaluate the effect of risk of bias; trials in which randomisation is inadequately concealed or inadequately reported are known to be empirically associated with exaggerated treatment effects [41]. In addition, we also tested, post-hoc, whether missing data impacted on the size and direction of effect sizes by running a sensitivity analysis that excluded studies that reported losses of >20% at follow-up or where losses to follow-up were unknown, and studies that either did not report using intention-to-treat, or where it was not possible to judge if intention-to-treat had been used.

We undertook a subgroup analysis to investigate treatment effects within four separate groups of interventions: cognitive and behaviour therapy (CBT), multi-component exercise training, self-management education, and relaxation. To determine whether severity of depression and/or anxiety were associated with the effectiveness of interventions, we investigated treatment effects in two population subgroups: 1) studies that included confirmed depressed and/or anxious samples or above threshold samples; and 2) studies where depression and/or anxiety severity was unknown at baseline.

In addition, we undertook, post-hoc, sub-group analysis to determine the effects of all non-exercise based interventions to enable comparison with effect sizes for the overall pooled analyses for both depression and anxiety.

## Results

### Characteristics of Populations

Thirty two studies met the inclusion criteria, and included 35 relevant comparisons, of which 30 (n = 2063) could be included in the meta-analysis (Figure S1). All included studies were individually randomised controlled trials. The COPD patients had a median age of 66.3 years; one study recruited a male only sample (median 59% male). The majority of studies recruited patients with moderate [33]–[36], [42]–[48] or severe COPD [38], [49]–[60]; one study recruited patients with mild to moderate COPD [37], and in three studies patients in the intervention group had milder disease than patients in the control group, but this did result in baseline imbalance [32], [61], [62]. Only a minority of studies (*k* = 5) recruited patients with identified depression and anxiety [37], [44], [50], [51], [56]. In addition, using established cut-offs, seven studies reported baseline mean scores indicative of mild depression and four studies reported baseline mean scores indicative of mild anxiety. The average length of follow-up at post-treatment was 10.5 weeks (range 4 to 52 weeks). See Table 2 for population characteristics.

**Table 2:** Characteristics of the study populations.

Study	Sample size	Mean age	Males (%)	COPD severity (GOLD stage)	Where recruited	Depressed at baseline?	Anxious at baseline?	Depression assessment	Anxiety assessment	Baseline mean (SD) depression score		Baseline mean (SD) anxiety score
Blumenthal et al 2006	158	50	44	Severe (stage 3)	Secondary care	No	No	BDI	STAI	I = 13.4 (8.3); C = 10.9 (7.4)		I = 40.3 (12.6); C = 35.6 (11.3)
Bucknall et al 2012	464	69.1	37	I = severe (stage 3); C = severe (stage 3)	Secondary care	Yes	Yes	HADS	HADS	I = 8.5 (3.9); C = 8.3 (4.1)		I = 10 (4.5); C = 9.3 (4.6)
de Blok et al 2006	21	64.1	43	I = moderate (stage 2); C = severe (stage 3)	Tertiary care	No	No	BDI	N/A	I = 12.6 (95% CI 7.5–17.7); C = 12.9 (95% CI 8.5–17.2)		N/A
de Godoy and de Godoy 2003	30	60.5	73	Severe (stage 3)	Secondary care	Yes	Yes	BDI	BAI	I = 13.7 (8.9); C = 14.9 (11.5)		I = 12.9 (6.9); C = 10.9 (9.8)
Donesky-Cuenco et al 2009	41	70	28	I = moderate (stage 2); C = severe (stage 3)	Primary care	No	No	CES-D	STAI	I = 9.5 (4.5); C = 12.6 (9.4)		I = 30.2 (8); C = 33.8 (9)
Effing et al 2009	142	63.4	59	I = moderate (stage 2); C = severe (stage 3)	Secondary care	No	No	HADS	HADS	I = 4.4 (3.5); C = 4.6 (4)		I = 4.6 (3.3); C = 4.8 (4)
Elçi et al 2008	78	58.9	85	Severe (stage 3)	Tertiary care	No	No	HADS	HADS	Not reported	Not reported	
Emery et al 1998	79	66.6	47	Severe (stage 3)	Primary care	No	No	SCL-Depression	SCL-Anxiety	I (a) = 59.2 (7.6); I (b) = 55.5 (5.3); C = 60 (7.7)	I (a) = 54.3 (7.2); I (b) = 54.0 (5.3); C = 53.4 (4.5)	
Gift et al 1992	26	68.5	31	Moderate (stage 2)	Primary care	No	No	N/A	STAI	N/A	I = 45 (9); C = 37 (6)	
Griffiths et al 2000	200	68.3	60	Severe (stage 3)	Primary care and secondary care	No	No	HADS	HADS	I = 7.3 (3.2); C = 7.5 (4.3)	I = 8.6 (4.7); C = 8.9 (4.3)	
Güell et al 2006	40	67	94	Severe (stage 3)	Tertiary care	No	No	SCL-90-R	SCL-9-R	I = 1.3 (0.8); C = 0.6 (0.6)	I = 1.0 (0.5); C = 0.6 (0.7)	
Hospes et al 2009	39	62.2	60	Moderate (stage 2)	Secondary care	No	No	BDI	N/A	I = 8.4 (5.2); C = 9.1 (8.3)	N/A	
Hynninen et al 2010	51	61	49	Moderate (stage 2)	Secondary care	Yes	Yes	BDI-II	BAI	I = 20.7 (8.6); C = 20.5 (9.7)	I = 17.5 (7.3); C = 17.5 (9.5)	
Kapella et al 2011	23	63	83	I = moderate (stage 2); C = moderate (stage 2)	Community	Unknown	Unknown	POMS-D	POMS-A	I = 9.9 (10.3); C = 10.4 (8.2)	I = 9.4 (8.2); C = 8.6 (3.7)	
Kayahan et al 2006	45	66	87	Moderate (stage 2)	Tertiary care	No	No	HAM-D	HAM-A	I = 5.43 (4.8); C = 7.18 (6.5)	I = 8.91 (6.9); C = 7.91 (6.6)	
Kunik et al 2001	53	71.3	83	Severe (stage 3)	Secondary care	No	No	GDS	BAI	I = 11.5 (.3); C = 7.7 (5.4)	I = 15.3 (9.2); C = 10 (6.8)	
Kunik et al 2008	238	66.3	97	Severe (stage 3)	Primary care	Yes	Yes	BDI-II	BAI	I = 23.4 (12.5); C = 21.1 (12)	I = 22.67 (14.2); C = 23 (13.9)	
Lamers et al 2010	187	71	60	Mild to moderate (stage 1 to 2)	Primary care	Yes	No	BDI-II	SCL	I = 17.1 (6.5); C = 18.3 (7.2)	I = 20.6 (6.2); C = 20.4 (7.3)	
Livermore et al 2010	41	73.4	44	Moderate (stage 2)	Secondary care	No	No	HADS	HADS	I = 3.9 (2.1); C = 4.1 (2.8)	I = 5.2 (2.9); C = 5.9 (2.7)	
Lolak et al 2008	83	67.7	37	Severe (stage 3)	Secondary care	No	No	HADS	HADS	I = 6.6 (4); C = 4.9 (3)	T = 6 (4.3); C = 6.35 (3.8)	
Lord et al 2010	28	67.4	Not stated	Severe (stage 3)	Secondary care	No	No	HADS	HADS	I = 5.7 (2.8); C = 5.8 (3.6)	I = 6.3 (3.1); C = 5.3 (2.6)	
McGeoch et al 2006	159	71	59.5	Moderate (stage2)	Primary care	No	No	HADS	HADS	I = 4.6 (3.7); C = 4.1 (2.9)	I = 6.2 (4.2); C = 5.3 (3.6)	
Özdemir et al 2010	50	62.5	100	Moderate (stage 2)	Tertiary care	No	No	HADS	HADS	I = 6 (3); C = 7.0 (4.6)	I = 6.8 (3.2); C = 7.1 (4.9)	
Paz-Díaz et al 2007	24	64.5	73	Severe (stage 3)	Secondary care	No	No	BDI	STAI	I = 14 (8); C = 18 (8)	I = 35 (26); C = 33 (25)	
Ries et al 1995	119	62.6	73	Severe (stage 3)	Primary care	No	No	CES-D	N/A	I = 14.0 (8.7); C = 15.3 (10)	N/A	
Sassi-Dambron et al 1995	89	67.4	55	Moderate (stage2)	Secondary care	No	No	CES-D	STAI	I = 14.2 (10.2); C = 11.9 (7.6)	I = 33.8 (9.7); C = 34.1 (9.5)	
Spencer et al 2010	59	66	46	Moderate (stage 2)	Secondary care	No	No	HADS	HADS	I = 4 (2); C = 5 (3)	I = 6 (3); C = 6 (3)	
Taylor et al 2009	116	69.5	46	Moderate (stage 2)	Primary care	No	No	HADS	HADS	I = 5.4; C = 4.8	I = 6.1; C = 6.7	
Yeh et al 2010	10	65.5	60	Moderate (stage 2)	Secondary care	No	No	CES-D	N/A	I = 14 (11–46); C = 12 (2–17) (median, range)	N/A	

BAI = Beck Anxiety Inventory; BDI = Beck Depression Inventory; CES-D = Centre for Epidemiologic Studies Depression Scale; C = Control group; GOLD = Global initiative for chronic Obstructive Lung Disease; HADS = Hospital Anxiety and Depression Scale; HAM-A = Hamilton Anxiety Rating Scale; HAM-D = Hamilton Depression Rating Scale; I = Intervention group; N/A = not applicable; POMS-A = Profile of Mood States Anxiety scale; POMS-S = Profile of Mood States Depression scale; SCL-90 = Symptom Checklist-90; SD = Standard deviation; STAI: State Trait Anxiety Inventory.

### Characteristics of Interventions

The content, duration, intensity and delivery of the interventions varied considerably between the included trials. Over half (65%) of the interventions included both psychological and lifestyle components, while six included only psychological components [35], [42], [44], [45], [55], [56], and four focused on lifestyle alone [34], [38], [46], [48]. The most common psychological components were cognitive and behavioural interventions, problem solving techniques, relaxation and miscellaneous stress management interventions. The most common lifestyle components were structured exercise training, skills training, and education, typically as part of a pulmonary rehabilitation programme. The average number of treatment contacts (including remote contacts) was 18 (range 1 to 63), and the length of treatment sessions ranged from 30 to 240 minutes (mean 81.5 hours). Mean length of interventions was 11 weeks. A wide range of professionals and para-professionals (e.g. lay trainers) delivered the interventions, with the majority delivered face to face, either in groups or to individuals (see Table 3 for characteristics of interventions).

**Table 3:** Characteristics of the interventions.

Author	Intervention	Control group	Lifestyle components	Psychological components	No. Sessions	Session length (minutes)	Delivered by	Delivery method	Follow-up
Blumenthal et al 2006	Telephone-based coping skills training	Usual medical care including clinic visits with the pulmonologists and regular contact with the nurse coordinators.	General education; Relapse prevention	Problem-solving techniques; CBT; Relaxation	12	30	Clinical psychologists social workers	Individual, face-to-face, and remote.	12 weeks
Bucknall et al 2012	Supported self-management	Usual medical care from GP and hospital based specialists (including out of hours emergency care)	General education; Skills training	Miscellaneous (self-efficacy)	22	40	Respiratory nurses	Individual, face-to-face	52 weeks
de Blok et al 2006	PR plus physical activity counselling	Regular PR containing exercise training, dietary intervention and educational modules.	General education; Exercise; Skills training; Behaviour therapy	Biofeedback; Miscellaneous (Physical activity counselling, motivational interviewing)	4	30	Physical therapists	Group and individual, face-to-face	9 weeks
de Godoy and de Godoy 2003	CBT, physiotherapy, exercise and education	Physiotherapy, exercise and education	General education; Exercise; Skills training	CBT; Relaxation; Miscellaneous (Logotherapy)	24 exercise sessions; 24 physio sessions; 12 psycho-therapy sessions	Not reported	Respiratory physicians	Group, face-to-face	12 weeks
Donesky-Cuenco et al 2009	Yoga training	Usual care (also received educational pamphlet, offered yoga at the end as waiting list control)	Exercise; Skills training	Miscellaneous (relaxation)	24	60	Expert yoga instructors	Group, face-to-face	12 weeks
Effing et al 2011	Psycho-therapeutic exercise; self-management education	Self-management education.	General education; Skills training; Exercise	Problem-solving techniques	4 education sessions; First phase: 72 exercise sessions; Second phase: 40 voluntary exercise sessions	120 education sessions	Respiratory nurse and physio-therapist	Group, face-to-face, and remote.	28 weeks
Elçi et al 2008	PR	Standard medical care (including instructions on the use of respiratory medicines).	General education; Exercise; Skills training	Miscellaneous (psychological counselling)	24	90	Nurse	Individual, face-to-face, and remote	4 weeks
Emery et al 1998	Treatment (a) = Exercise, education and stress management Treatment (b) = education and stress management	Waiting list control	General education; Group discussion; Exercise	CBT; Relaxation; Miscellaneous (stress management)	37 exercise classes; 16 lectures; 10 stress management sessions	240 (all modules)	Respiratory specialists and clinical psychologist	Group, face-to-face	10 weeks
Gift et al 1992	Progressive muscle relaxation with pre-recorded tapes	Participants instructed to sit quietly for 20 minutes	N/A	Relaxation (Bernstein and Borkovec method)	4	20	Primary care practitioners	Individual, face-to-face	4 weeks
Griffiths et al 2000	Multi-disciplinary PR	Standard medical management	General education; Exercise; Skills training	Relaxation; Miscellaneous (stress management to promote mastery and control over illness)	18	120	Occupational therapist physio-therapist, dietetic staff, specialist respiratory nurse and a smoking-cessation counsellor.	Group, face-to-face	6 weeks
Güell et al 2006	PR including breathing training and exercise	Usual care	General education; Exercise; Skills training	Relaxation	Phase 1 = 16 sessions; Phase 2 = 40 sessions	30	Not reported	Group, face-to-face	16 weeks
Hospes et al 2009	Pedometer-based exercise counselling programme	Usual care	Exercise	Biofeedback; Problem-solving techniques; Exercise counselling; Motivational interviewing	5	30	Trained exercise counsellor	Individual, face-to-face	12 weeks
Hynninen et al 2010	CBT	Enhanced standard care for COPD	N/A	CBT	7	60	Masters level psychology student	Group, face-to-face	4 weeks
Kapella et al 2011	CBT	COPD education	N/A	CBT	6	Not reported	Nurse behavioural sleep medicine specialist	Group, face-to-face	6 weeks
Kayahan et al 2006	PR	Usual care	General education; Exercise; Skills training	Relaxation	24	150	Not reported	Individual and group, face-to-face	8 weeks
Kunik et al 2001	CBT	COPD education	N/A	CBT	1 (+6 phone calls)	120	Board-certified gero-psychiatrist	Group, face-to-face and individual, remote	6 weeks
Kunik et al 2008	CBT group treatment intervention	COPD education	N/A	CBT	8	60	Psychology interns and postdoctoral fellows	Group, face-to-face	4 weeks
Lamers et al 2010	Minimal psychological intervention	Usual care	Skills training	Problem-solving techniques; CBT	Average of 4 contacts	60	Primary care nurses	Individual, face-to-face	12 week
Livermore et al 2010	CBT	Routine care (including PR)	N/A	CBT	4	60	Clinical psychologist	Individual, face-to-face	6 weeks
Lolak et al 2008	Progressive muscle relaxation and PR	Exercise training	General education; Exercise; Skills training	Relaxation (Bernstein and Borkovec method)	12	60	Multi-disciplinary PR team	Group, face-to-face	8 weeks
Lord et al 2010	Singing teaching	Usual care	Skills training	Relaxation	12	60	Singing teacher	Group, face-to-face	7 weeks
McGeoch et al 2006	Usual care and education on the use of a written self-management plan.	Usual GP care	General education; Skills training	N/A	1	60	Practice nurse or respiratory educator in association with GP	Individual, face-to-face	24 weeks
Özdemir et al 2010	Water-based PR	Usual care	Exercise	N/A	12	35	Physio-therapist and chest physician.	Group, face-to-face	4 weeks
Paz-Díaz et al 2007	Exercise rehabilitation programme	Usual care.	Exercise; Skills training	Miscellaneous (relaxation techniques)	24	85	Not reported	Group, face-to-face	8 weeks
Ries 1995	Pulmonary rehabilitation	Education (videotapes, lectures, and discussions but no individual instruction or exercise training)	General education; Exercise; Skills training	Relaxation; Miscellaneous (psychological support)	12	240	Not reported	Group, face-to-face	8 weeks
Sassi-Dambron et al 1995	Dyspnoea self-management training	General health education.	General education; Group discussion; Skills training	Relaxation (progressive muscle relaxation); Miscellaneous (self-talk and panic control)	6	not reported	Graduate student in psychology and a clinical nurse	Group, face-to-face	6 weeks
Spencer et al 1995	Supervised outpatient-based exercise plus unsupervised home exercise	Unsupervised exercise	Exercise	N/A	52	50	Physio-therapist	Group, face to face	12 weeks
Taylor et al 2009	Disease-specific self-management programme	Usual care	Skills training	Miscellaneous (Self-management using social cognitive self-efficacy theory)	7	150	Lay trainer and respiratory physician	Group, face-to-face	8 weeks
Yeh et al 2010	Tai Chi classes	Usual care	Exercise	Relaxation; Miscellaneous (meditation and mindfulness)	24	60	Tai Chi instructors	Group, face-to-face	12 weeks

CBT = Cognitive and Behavioural Therapy; GP = General Practitioner; N/A = Not applicable; PR = Pulmonary rehabilitation.

### Risk of Bias

**Figure 1:**
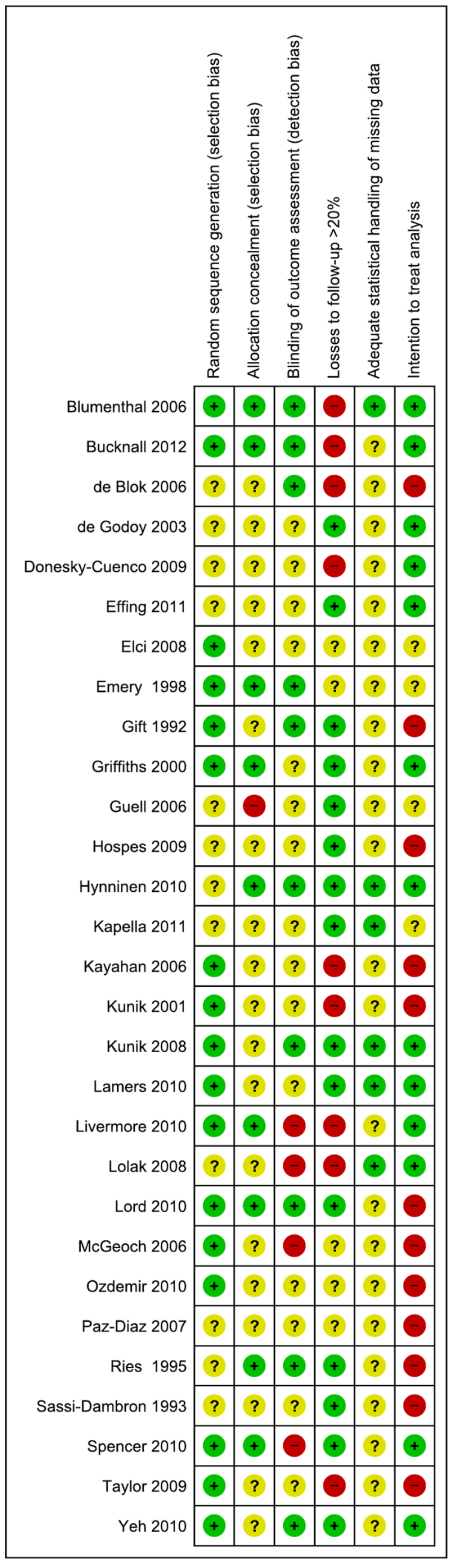
Risk of bias summary: review authors’ judgements about each risk of bias item for each included study.

Seventeen (59%) of the 29 trials described an adequate method of random sequence generation, but only nine reported adequate methods of allocation concealment; the method of allocation concealment was unclear in 19 (65%) trials, and one trial did not conceal treatment allocation (Figure 1). Blinding of outcome assessors for anxiety and depression outcomes was reported in ten trials (34%). Losses to follow-up of >20% occurred in nine (31%) trials but only two of these reported using statistical methods (full information maximum likelihood method and substitution of baseline scores) to replace missing values at follow-up. Thirteen (45%) trials stated that an intention-to-treat approach was used.

### Meta-analysis

**Figure 2:**
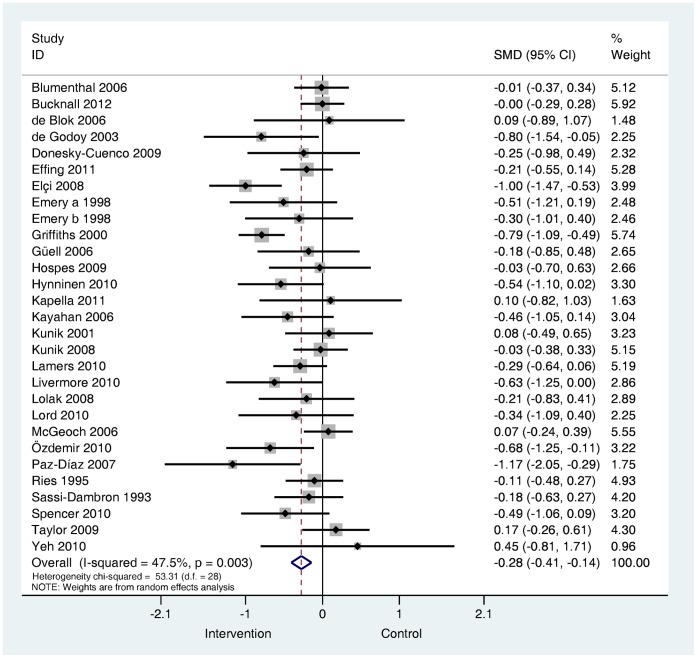
Effects of complex interventions on self-reported depression symptoms at post-treatment. Note: Meta-analysis of Individual trial and pooled effects. Random effects model used. 95% CI = 95% confidence intervals; SMD = standardised mean difference.

**Figure 3:**
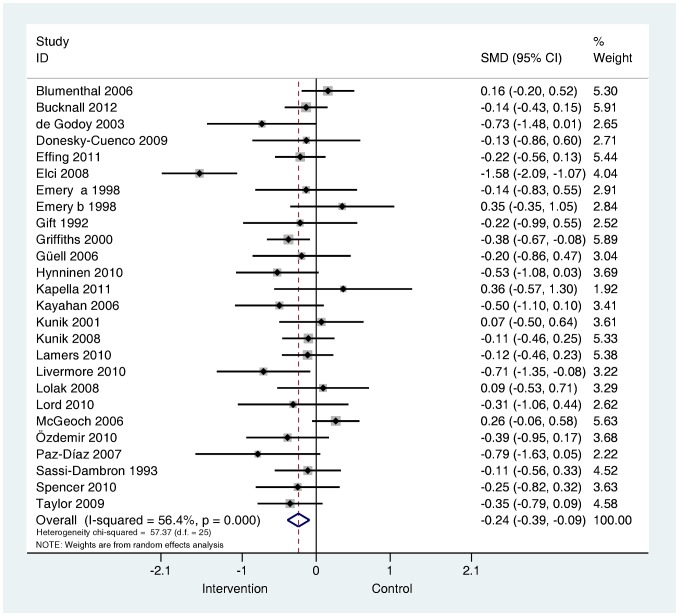
Effects of complex interventions on self-reported anxiety symptoms at post-treatment. Note: Meta-analysis of individual trial and pooled effects. Random effects model used. 95% CI = 95% confidence intervals; SMD = standardised mean difference.

**Are psychological and lifestyle interventions effective in reducing symptoms of anxiety and depression in patients with COPD?** Depression was reported in 29 trials and anxiety was reported in 26 trials. Interventions were associated with small, significant improvements in depression (SMD −0.28, 95% confidence interval −0.41 to −0.14, I^2^ = 47.5%, P = 0.003; Figure 2) and in anxiety (SMD −0.24, 95% confidence interval −0.39 to −0.09, I^2^ = 56.4%, P = 0.000; Figure 3).

### Small Study Bias

**Figure 4:**
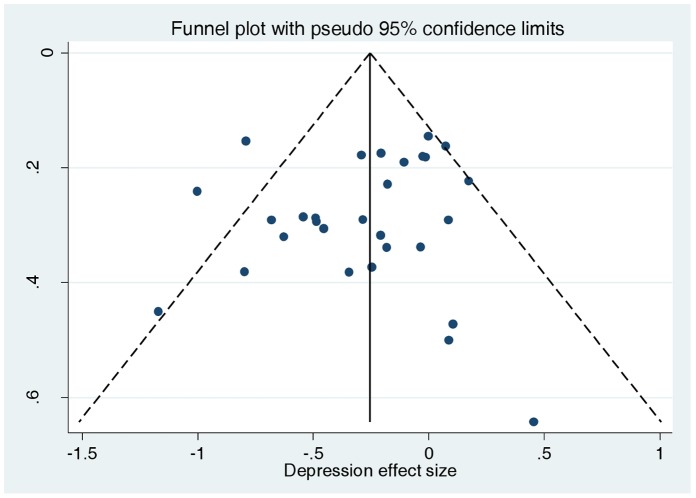
Funnel plot of effect size versus standard error for depression outcomes.

**Figure 5:**
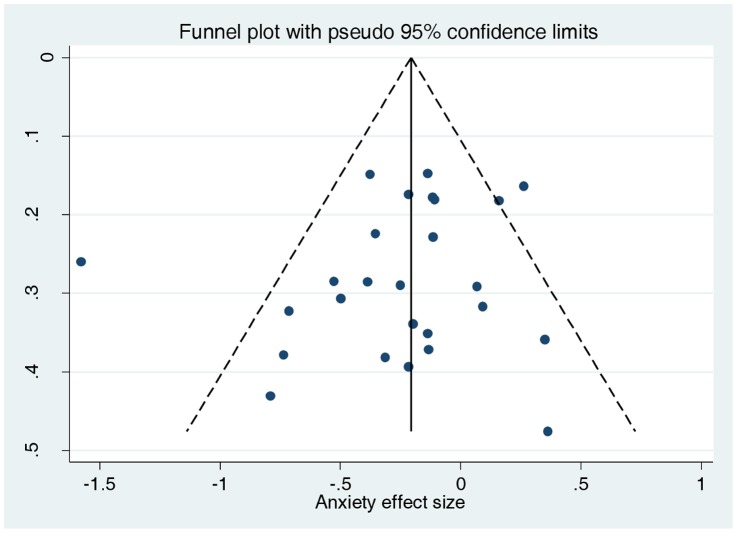
Funnel plot of effect size versus standard error for anxiety outcomes.

We found no evidence of funnel plot asymmetry for either depression (Egger test P = 0.413; Figure 4) or anxiety (Egger test P = 0.295; Figure 5).

### Sensitivity Analysis

**Does risk of bias impact the size and direction of treatment effects?** We removed from the pooled analyses for depression and anxiety studies in which the method of allocation concealment was either not reported or was inadequate. For depression, nine studies remained in the meta-analysis; the magnitude of the effect size increased marginally compared with the larger pooled analysis (SMD −0.32, 95% confidence interval −0.56 to −0.08, I^2^ = 59%, P = 0.01). For anxiety, eight studies remained in the meta-analysis, resulting in a small reduction of the effect size compared with the larger pooled analysis (SMD −0.21, 95% confidence interval −0.40 to −0.02, I^2^ = 33%, P = 0.16).

In a separate sensitivity analysis we also removed from the pooled analyses for depression and anxiety studies which reported losses of >20% at follow-up or where losses to follow-up were unknown, and studies that either did not report using intention-to-treat, or where it was not possible to judge if intention-to-treat had been used. For depression, 17 studies remained in the meta-analysis resulting in a small reduction in the effect size, and lower but still significant heterogeneity compared with the larger pooled analysis (SMD −0.26, 95% confidence interval −0.41 to −0.12, I^2^ = 39.3%, P = 0.049). For anxiety, 11 studies remained in the meta-analysis, resulting in a small reduction in the effect size and non-significant heterogeneity compared with the larger pooled analysis (SMD −0.20, 95% confidence interval −0.35 to −0.05, I^2^ = 21.2%, P = 0.242).

### Subgroup Analyses

**Figure 6:**
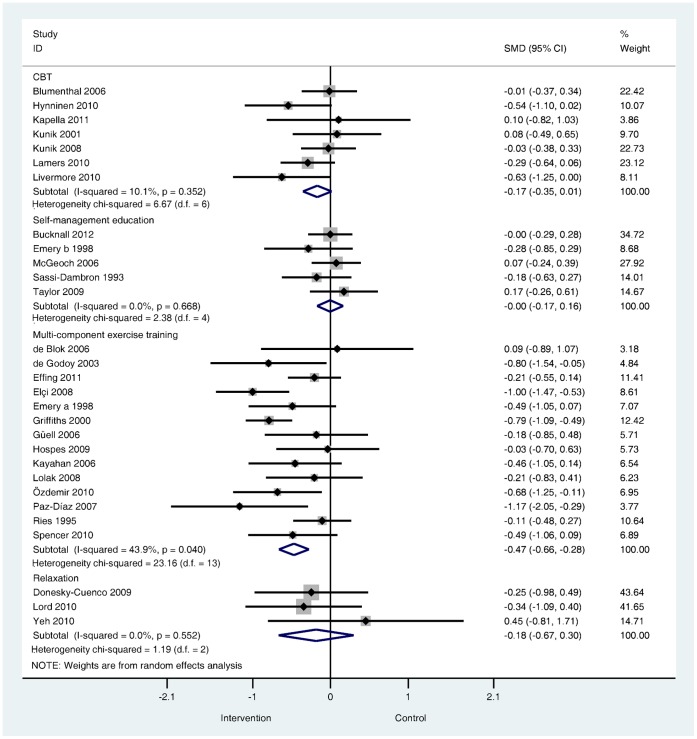
Effects of complex interventions by sub-group on self-reported symptoms of depression at post-treatment. Note: Random effects model used. 95% CI = 95% confidence interval; SMD = standardised mean difference.

**Figure 7:**
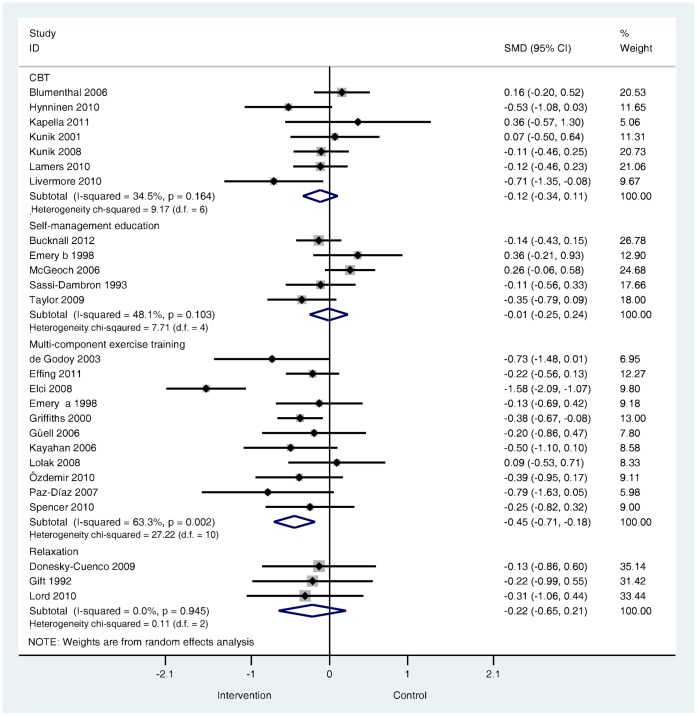
Effects of complex interventions by sub-group on self-reported symptoms of anxiety at post-treatment. Note: Random effects model used. 95% CI = 95% confidence interval; SMD = standardised mean difference.

**What types of psychological and lifestyle interventions are most effective?** The direction and magnitude of effect sizes were similar across the four intervention subgroups for both depression and anxiety outcomes (Figure 6 and Figure 7). The subgroup of trials that used multi-component exercise training [32], [34], [43], [45], [48], [51]–[54], [57], [59], [60], [62] were associated with moderate and significant effects but exhibited moderate to substantial heterogeneity (for depression I^2^ = 43.9%, P = 0.040; for anxiety I^2^ = 63.3%, P = 0.002). Small but non-significant effects were observed in the subgroup of trials that tested relaxation techniques [33], [42], [58], [61]. Similarly, the subgroup of trials that tested CBT [35], [37], [44], [49], [55], [56], [63] were associated with the small, non-significant treatment effects. The subgroup that tested self-management education [36], [46], [47], [50], [52] were associated with no treatment differences between intervention and control groups for either depression or anxiety.

**Figure 8:**
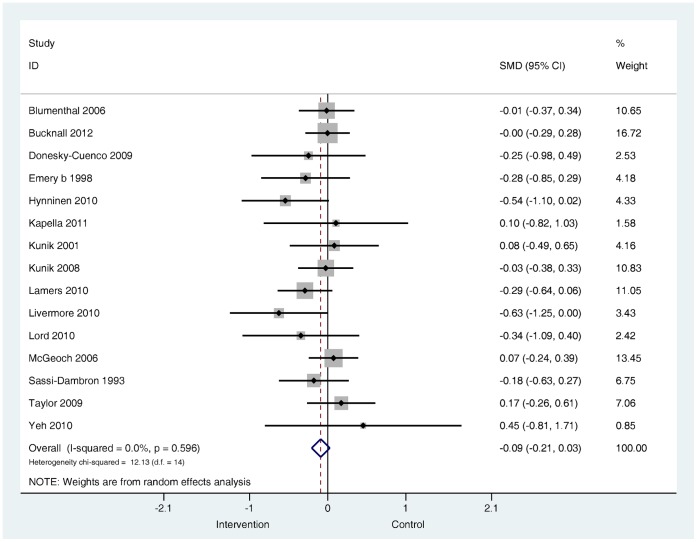
Effects on self-reported symptoms of depression in sub-group of non-exercise based complex interventions. Note: Random effects model used. 95% CI = 95% confidence interval; SMD = standardised mean difference.

**Figure 9:**
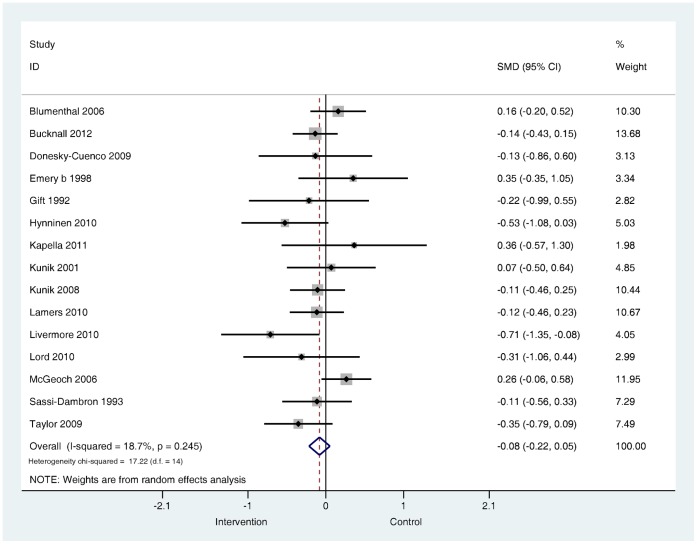
Effects on self-reported symptoms of anxiety in sub-group of non-exercise based complex interventions. Note: Random effects model used. 95% CI = 95% confidence interval; SMD = standardised mean difference.

When studies that only tested non-exercise based complex interventions were pooled in a sub-group the overall results for depression (*k* = 17) and for anxiety (*k* = 11) favoured the intervention, but were non-significant (Figure 8 and Figure 9).

**Figure 10:**
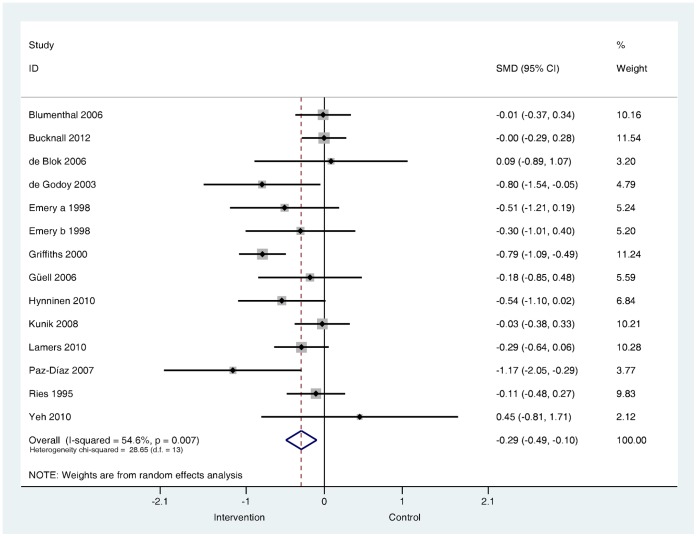
Effects on self-reported symptoms of depression in trials that included confirmed depressed samples or above threshold samples. Note: Random effects model used. 95% CI = 95% confidence intervals; SMD = standardised mean difference.

**Figure 11:**
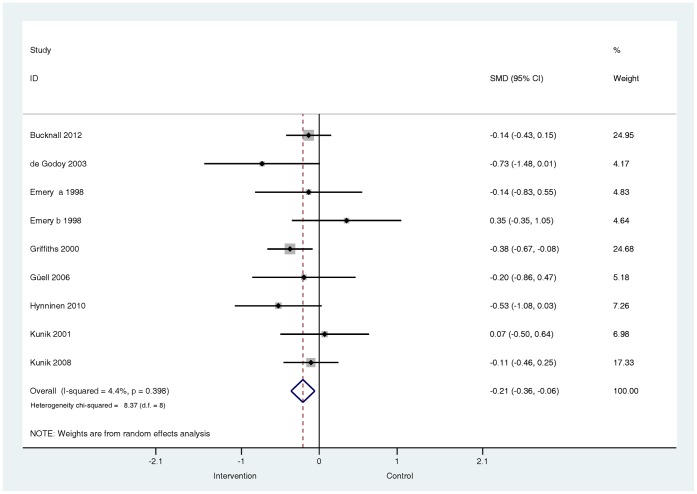
Effects on self-reported symptoms of anxiety in trials that included confirmed anxious samples or above threshold samples. Note: Random effects model used. 95% CI = 95% confidence intervals; SMD = standardised mean difference.

**Figure 12:**
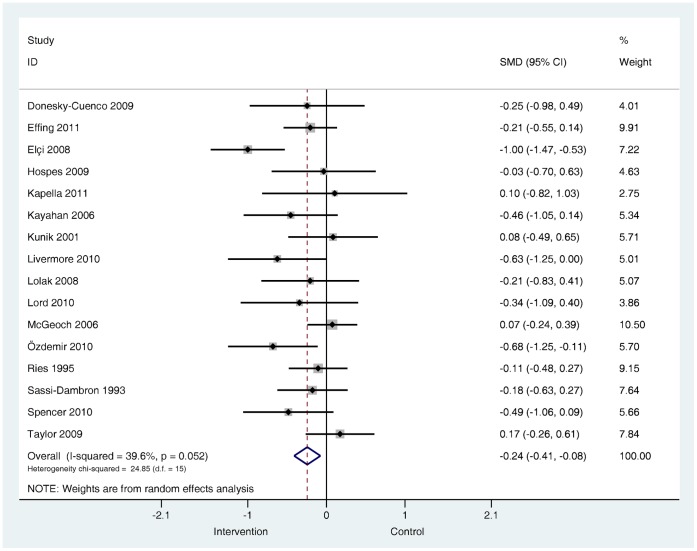
Effects on self-reported symptoms of depression in trials where severity of depression was unknown at baseline. Note: Random effects model used. 95% CI = 95% confidence intervals; SMD = standardised mean difference.

**Figure 13:**
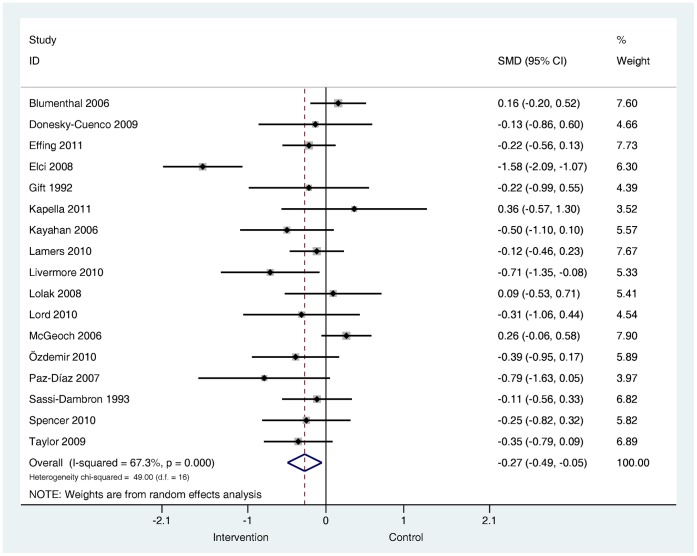
Effects on self-reported symptoms of anxiety in trials where severity of anxiety was unknown at baseline. Note: Random effects model used. 95% CI = 95% confidence intervals; SMD = standardised mean difference.

**Do these effects vary by patient population?** In the subgroup of studies (*k* = 13) that included confirmed depressed or above threshold samples the effects were very similar to the pooled effects from the larger group of treatment comparisons, (SMD −0.29, 95% confidence interval −0.49 to −0.10, I^2^ = 54.6%, P = 0.007) (Figure 10). The effects of the subgroup of studies (*k* = 8) that included samples of confirmed anxious and above threshold samples were, like the pooled effects from the larger group of comparisons, small, but significant (SMD −0.21, 95% confidence interval −0.36 to −0.03, I^2^ = 4.4%, P = 0.398) (Figure 11). Small, significant treatment effects were also observed for depression outcomes (SMD −0.24, 95% confidence interval −0.41 to −0.08, I^2^ = 39.6%, P = 0.052) and anxiety outcomes (SMD −0.27, 95% confidence interval −0.49 to −0.05, I^2^ = 67.3%, P = 0.000) in the subgroup of studies that included samples where depression (*k* = 16) and anxiety (*k* = 17) severity was unknown at baseline (Figure 12 and Figure 13).

## Discussion

### Main Findings

This meta-analysis of 29 trials (30 comparisons) shows that complex psychological and/or lifestyle interventions that include exercise components are associated with moderate and significant treatment effects over the short term compared with usual care or active control groups. Small, non-significant treatment effects were found for the subgroups of trials that tested CBT and relaxation techniques. No significant difference in treatment effects were found for trials that compared self-management education with a control group. Overall, treatment effects were not substantially different in the subgroup of studies that included confirmed depressed and/or anxious samples or above threshold samples, and the subgroup of studies that included samples where severity of depression and/or anxiety was unknown.

### Strengths and Limitations

This systematic review used rigorous search methods to identify all randomised controlled trials of complex interventions that included psychological and/or lifestyle components and measured depression and/or anxiety across a broad range of severity in people with COPD. Included trials differed significantly in terms of interventions, patient populations, study quality and follow-up times, which limits the extent to which broad conclusions can be made about the overall effectiveness of complex interventions for depression and anxiety in COPD populations.

However, we increased homogeneity by standardising follow-up times across outcomes and estimates of heterogeneity in the pooled analyses were moderate by conventional thresholds [39]. Moreover, there is a strong argument for avoiding narrow approaches to meta-analysis and to instead adopt broad and inclusive approaches that maximise power and allow exploratory (subgroup) analyses – meta-analyses can tolerate substantial clinical and methodological heterogeneity and publication bias and study quality may be a more important threats to validity of results [64]. We therefore tested for publication bias and investigated whether selection bias owing to inadequate allocation concealment exaggerated treatment effects for both outcomes. In addition we undertook a small series of pre-planned subgroup analyses to determine the relative effects of different interventions. While this approach might provide more useful data to support design and delivery of the most effective interventions for managing depression and anxiety in COPD, such subgroup analyses should be interpreted cautiously because other, unanalysed differences between studies might account for the results [65].

The optimal strategy to explore heterogeneity is to therefore use meta-regression which investigates whether particular covariates (potential ‘effect modifiers’) explain any of the heterogeneity of treatment effects between studies [66]. However, the results of meta-regression only represent observational not causal associations and lack power in the presence of small sample sizes. In addition, because the vast majority of complex intervention trials are still not designed, conducted or reported in line with the UK Medical Research Council Framework [67] it is difficult to isolate the active ingredients of interventions such as those included in this review using meta-regression, leading to calls to strengthen and improve the reliability of specifications of characteristics of behaviour change interventions [68].

It is plausible that the small effects of the overall pooled analyses reflect that only a subset of trials included in this review recruited patients with confirmed depression and/or anxiety. However, compared with the larger pooled analyses, treatment effects were not substantially larger in the subgroup of trials that included samples of confirmed depressed and/or anxious patients or above threshold samples. Furthermore, small, significant treatment effects were associated with the subgroup of trials that included samples where the severity of depression and/or anxiety status was not known. These results suggest that all trials in this review will have included patients with symptoms of depression and/or anxiety, including sub-threshold symptoms, which are known to affect health outcomes in long term conditions and should be proactively managed [69].

Finally, because the meta-analysis was based on post-intervention sample sizes it is possible that the results do not reflect intention-to-treat analyses but rather available case analyses. Even in those studies that imputed missing data using conventional methods, the final sample sizes may not truly reflect intention-to-treat analyses [70]. However, in the absence of raw individual patient data the meta-analyst is therefore reliant on using data from participants whose results are known and should instead address the potential impact of missing data through risk of bias assessments [65]. We followed this advice by performing a sensitivity analysis to exclude studies where intention-to-treat was not used or reported and where losses to follow up exceeded 20%. While there appeared to be no clinically relevant differences in observed effect sizes between this sensitivity analysis and the overall pooled analysis the presence or absence of an intention-to-treat approach and losses to follow-up>20% may well account for significant amounts of heterogeneity, as indicated by the changes we observed in the significance tests for heterogeneity.

Additionally, three of the eligible studies did not contain meta-analysable data and attempts to contact study authors failed to recover usable data [71]–[73].

### Implications for Research and Practice

The overall results of this review are in-keeping with those of comparable comprehensive reviews of the effectiveness of complex psychological and lifestyle interventions on depression in people with diabetes [26] and coronary heart disease [74]. All three reviews have shown that overall, complex psychological and/or lifestyle intervention reduce depression but the effects are small. However, our review differs from those by Harkness et al and Dickens et al because we have shown that the small but significant effects observed for the pooled analyses for both depression and anxiety outcomes is largely driven by the inclusion of positive trials that tested exercise interventions. Indeed, multi-component exercise training with or without psychological components, and ordinarily given as part of pulmonary rehabilitation, were the only sub-group of interventions that significantly reduce both depression and anxiety; this remained true even when outliers were removed from the analyses [38], [59]. When expressed as a binomial effect the estimate of effect of multi-component exercise training on depression is equivalent to a clinical effect of 22% beyond chance, potentially benefiting 193 of the 878 patients included in this subgroup of trials in this review. In terms of absolute risk reduction, this is equivalent to a number needed to treat of 5– reductions in symptoms of depression would be expected in 1 in 5 (95% confidence interval 3.5 to 6.4) patients exposed to multi-component exercise training.

When expressed as a binomial effect, the effect of multi-component exercise training on anxiety is less than for depression, equivalent to a clinical effect of 9.8% beyond chance, potentially benefiting 73 of the 719 patients in this subgroup of trials included in this review. This equates to a number needed to treat of 11– reductions in symptoms of anxiety would be expected in 1 in 11 (95% confidence interval 5.8 to 38.5) patients exposed to multi-component exercise training.

Exercise may offer patients an alternative and effective approach to managing depression, especially for those with physical comorbidities who have concerns about the antagonistic effects and burden of multiple medications [75]. However the science underpinning the use of exercise to treat depression is uncertain and even less robust in relation to anxiety. Based on high quality trials alone a Cochrane review has shown that exercise can improve depression in adults without long term conditions, and is as effective as cognitive therapy, but the effects are small [76]. By comparison two large meta-analyses concluded that exercise training, undertaken for 30 minutes for 3 to 12 weeks has positive effects on depression and anxiety in people with long term conditions [77], [78]. While novel in their insights about the efficacy of exercise for depression and anxiety in chronically ill populations the reviews by Herring et al only included a small number of COPD trials and did not look at the comparative effectiveness of other psychological or lifestyle interventions. By contrast our review is the first to specifically demonstrate that structured exercise training significantly reduces both symptoms of depression and anxiety in people with COPD. Furthermore we have showed that structured exercise training is more effective than other, complex psychological behavioural and lifestyle interventions previously thought to improve mental health in people with COPD.

While this review therefore represents a considerable advance on understanding the positive role exercise training can play in managing depression and anxiety in COPD, its main finding appears at odds with the results of a facilitated exercise intervention which increased physical activity but did not reduce depression or use of anti-depressants over 12 months in primary care patients with recognised depression [79]. However, the negative and controversial results of the trial by Chalder et al can be partly be explained by the fact that patients in the two arms engaged in very similar levels of exercise. Additionally, it is likely that the overall effect size of this trial was diluted by heterogeneity of response among individuals and we do not fully understand the factors that relate to this heterogeneity [80]. Moreover, outside of the context of pulmonary rehabilitation, increasing physical activity in sedentary and depressed and/or anxious COPD patients remains a challenge. This is especially true in primary care, but advice and counselling are effective methods to promote increased physical activity for people with and without long term conditions [81], whereas the cost-benefits of exercise referral schemes are uncertain [82].

The finding that CBT is not as effective as exercise training for depression or anxiety in COPD is inconsistent with clinical guidelines that recommend using low intensity psychological interventions as first line therapy for treating depression and anxiety in people with long term conditions [11]. CBT has a similar efficacy profile to antidepressants for managing depression and anxiety disorders [83], but in this review we found that the effectiveness of CBT is possibly reduced in the presence of COPD. Methodological parameters might partly account for this finding as a number of included trials in this subgroup compared CBT with active controls [55], [56], [63]. Alternatively, compared with exercise, CBT places relatively high cognitive demands on patients and treatment response to this form of psychological therapy may be compromised among older adults with cognitive impairments in memory, attention, and executive function. Patients in the CBT trials included in this review were generally over 60 years old and their engagement with CBT could have thus been affected by the presence of cognitive impairments. In addition, there is consistent evidence that COPD, in both non-hypoxic and hypoxic individuals, is associated with cognitive dysfunction not ordinarily associated with other comorbidities [84], further suggesting that CBT may not be appropriate for COPD patients with significant cognitive impairments.

The negative results for the CBT sub-group in this review may also owe to the fact that CBT, which centres on challenging thoughts and setting behavioural goals, is difficult to accommodate by people with COPD whose ruminative thinking and avoidance behaviours are associated with real and meaningful symptoms, especially dyspnoea. While there is growing evidence that CBT can improve generalised anxiety disorder and global mental health in older adults with medical diagnoses [85], psychological interventions that promote an ‘accepting mode of response’ such as mindfulness might be more appropriate and effective for managing psychological distress in COPD patients, especially breathing related anxiety. Mindfulness based interventions are associated with longer term benefits on psychological health than stand alone relaxation interventions [86], but their usefulness in COPD is yet to be established [87].

The least effective interventions in this review were self-management education programmes with or without action plans, and case management. Self-management education in COPD can prevent hospital admissions but they have little effect on other important outcomes such as exacerbations, respiratory symptoms, medication use and exercise capacity [88], and their effect on anxiety and depression has not been specifically assessed. By contrast, case management approaches that draw on integrated and collaborative approaches such as the chronic care model have been shown to reduce depression and improve physical health in people with diabetes and coronary heart disease [89], but their effectiveness and safety in COPD is unknown [90]. Developing integrated and collaborative care models that enhance opportunities for therapeutic synergies between mental and physical health is a major priority of advanced health systems confronted with increased prevalence and burden of multimorbidity [91]. There is then scope to explore the effectiveness of collaborative care models in the management of COPD, specifically testing whether the integration of exercise training in such models can confer physical and mental health benefits in people with COPD.

### Conclusion

Complex psychological and/or lifestyle interventions that include exercise training reduce symptoms of depression and anxiety in people with COPD over the short term. Additionally, complex interventions that included exercise training, with or without psychological components, were associated with the largest treatment effects in people with COPD across a range of disease severity when compared with usual care or active control groups. We were not able to determine the optimal dose of exercise training but the most effective interventions in this subgroup typically comprised 3 to 12 weeks of ≥30 minute sessions of group based exercise training in the context of pulmonary rehabilitation. No other intervention subgroups, including CBT, were associated with significant treatment effects. Treatment effects were no larger in trials that included confirmed depressed and/or anxious samples or above threshold samples, than in trials where severity of depression and anxiety was unknown at baseline. In conclusion, exercise training can have adventitiously positive effects on psychological health in all COPD patients, even among those with sub-threshold levels of depression and anxiety.

## Supporting Information

Figure S1 **PRISMA flowchart.**
(TIF)

Appendix S1 **PRISMA checklist.**
(DOC)

Appendix S2 **Search strategies.**
(DOC)
