# Supplementary figures and images for: The Effect of Complex Interventions on Depression and Anxiety in Chronic Obstructive Pulmonary Disease: Systematic Review and Meta-Analysis
Source: PLoS One. 2013 Apr 5;8(4):e60532. doi: 10.1371/journal.pone.0060532 (PMC3621386; doi:10.1371/journal.pone.0060532)

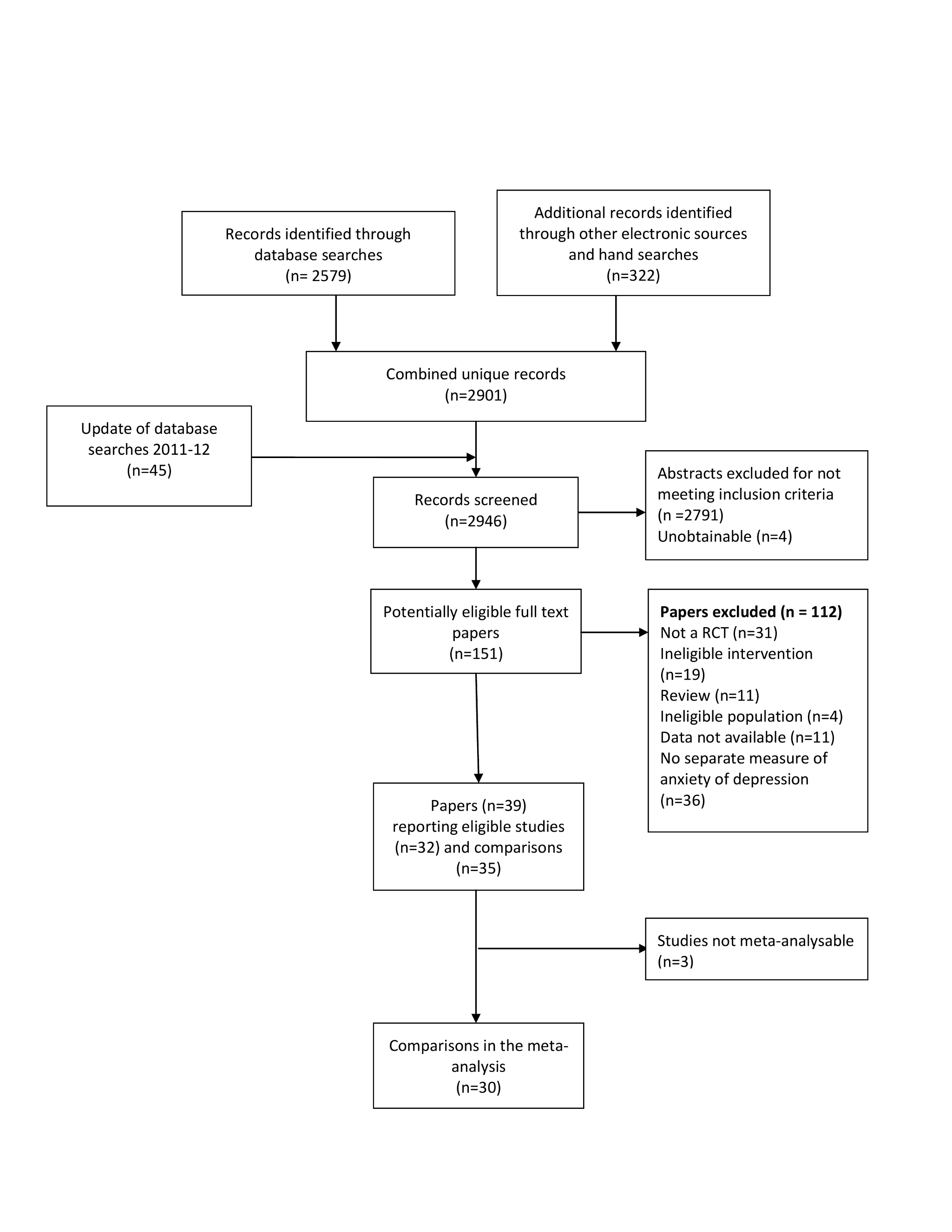

Supplement: Figure S1 — PRISMA flowchart. (TIF) [file pone.0060532.s001.tif]
