# Supplementary material for: The Effect of Complex Interventions on Depression and Anxiety in Chronic Obstructive Pulmonary Disease: Systematic Review and Meta-Analysis
Source: PLoS One. 2013 Apr 5;8(4):e60532. doi: 10.1371/journal.pone.0060532 (PMC3621386; doi:10.1371/journal.pone.0060532)
Supplement: Appendix S2 — Search strategies. (DOC) [file pone.0060532.s003.doc]

**Appendix S2**

**CENTRAL search**

1. MeSH descriptor Depression explode all trees
2. MeSH descriptor Depressive Disorder explode all trees
3. MeSH descriptor Affective Disorders, Psychotic explode all trees
4. MeSH descriptor Dysthymic Disorder explode all trees
5. MeSH descriptor Adjustment Disorders explode all trees
6. MeSH descriptor Depressive Disorder explode all trees
7. MeSH descriptor Anxiety explode all trees
8. MeSH descriptor Anxiety Disorders explode all trees
9. MeSH descriptor Mental Disorders explode all trees
10. MeSH descriptor Adaptation, Psychological explode all trees
11. MeSH descriptor Mental Health explode all trees
12. MeSH descriptor Mood Disorders explode all trees
13. MeSH descriptor Cognition Disorders explode all trees
14. MeSH descriptor Depressive Disorder, Major explode all trees
15. MeSH descriptor Panic Disorder explode all trees
16. MeSH descriptor Psychopathology explode all trees
17. MeSH descriptor Autogenic Training explode all trees
18. MeSH descriptor Counseling explode all trees
19. MeSH descriptor Cognitive Therapy explode all trees
20. MeSH descriptor Psychotherapy explode all trees
21. MeSH descriptor Relaxation Therapy explode all trees
22. MeSH descriptor Telephone explode all trees
23. MeSH descriptor Health Behavior explode all trees
24. MeSH descriptor Social Support explode all trees
25. MeSH descriptor Rehabilitation explode all trees
26. MeSH descriptor Adaptation, Psychological explode all trees
27. MeSH descriptor Cognitive Therapy explode all trees
28. MeSH descriptor Life Style explode all trees
29. MeSH descriptor Health Education explode all trees
30. MeSH descriptor Health Promotion explode all trees
31. MeSH descriptor Life Change Events explode all trees
32. MeSH descriptor Motivation explode all trees
33. MeSH descriptor Social Environment explode all trees
34. MeSH descriptor Self Care explode all trees
35. MeSH descriptor Meditation explode all trees
36. MeSH descriptor Anxiety explode all trees
37. MeSH descriptor Psychopathology explode all trees
38. MeSH descriptor Behavior Therapy explode all trees
39. MeSH descriptor Clinical Trials as Topic explode all trees
40. MeSH descriptor Placebo Effect explode all trees
41. MeSH descriptor Intervention Studies explode all trees
42. MeSH descriptor Program Evaluation explode all trees
43. (rct):ti,ab,kw
44. MeSH descriptor Pulmonary Disease, Chronic Obstructive explode all trees
45. MeSH descriptor Lung Diseases, Obstructive explode all trees
46. (Chronic airway obstruction):ti,ab,kw
47. (airflow limitation):ti,ab,kw
48. (chronic obstructive airway disease):ti,ab,kw
49. (chronic obstructive pulmonary disease):ti,ab,kw
50. (COPD):ti,ab,kw
51. (coa):ti,ab,kw
52. (pulmonary disease):ti,ab,kw
53. (dysthymia):ti,ab,kw
54. (reactive depression):ti,ab,kw
55. (major depression):ti,ab,kw
56. (masked depression):ti,ab,kw
57. (moderate depression):ti,ab,kw
58. (recurrent depression):ti,ab,kw
59. (long term depression):ti,ab,kw
60. (minor depression):ti,ab,kw
61. (mild depression):ti,ab,kw
62. (atypical depression):ti,ab,kw
63. (anxiety management):ti,ab,kw
64. (mental disease):ti,ab,kw
65. (psychological health):ti,ab,kw
66. (psychological stress):ti,ab,kw
67. (psychological distress):ti,ab,kw
68. (maladjustment):ti,ab,kw
69. (mental stress):ti,ab,kw
70. (low mood):ti,ab,kw
71. (health anxiety):ti,ab,kw
72. (psychiatric symptoms):ti,ab,kw
73. (mental distress):ti,ab,kw
74. (distress):ti,ab,kw
75. (coping):ti,ab,kw
76. (anxiety neurosis):ti,ab,kw
77. (neurosis):ti,ab,kw
78. (distress syndrome):ti,ab,kw
79. (neurotic disorders):ti,ab,kw
80. (coping behaviour):ti,ab,kw
81. (health anxiety):ti,ab,kw
82. (depressive symptoms):ti,ab,kw
83. (geriatric depression):ti,ab,kw
84. (psychological dysfunction):ti,ab,kw
85. (motivational interview):ti,ab,kw
86. (group therapy):ti,ab,kw
87. (health behaviour):ti,ab,kw
88. (support networks):ti,ab,kw
89. (nurse):ti,ab,kw
90. (counselling):ti,ab,kw
91. (pulmonary rehabilitation):ti,ab,kw
92. (psychotherapy):ti,ab,kw
93. (randomized):ti,ab,kw
94. (randomly):ti,ab,kw
95. (trial):ti,ab,kw
96. (controlled trial):ti,ab,kw

**Embase search**

1. exp RECURRENT BRIEF DEPRESSION/ or exp REACTIVE DEPRESSION/ or exp MAJOR DEPRESSION/ or exp DEPRESSION/ or exp ATYPICAL DEPRESSION/ or exp MASKED DEPRESSION/ or exp LONG TERM DEPRESSION/ or exp ORGANIC DEPRESSION/ or exp "MIXED ANXIETY AND DEPRESSION"/
2. depression.mp.
3. affective disorder.mp. or exp mood disorder/
4. affective disorder$.mp.
5. dysthymia.mp. or exp DYSTHYMIA/
6. dysthymic disorder.mp.
7. depressive disorder.mp.
8. exp major depression/ or recurrent depression.mp.
9. exp depression/
10. exp ANXIETY/ or exp ANXIETY DISORDER/
11. exp health anxiety/ or health anxiety.mp.
12. anxiety management.mp.
13. mental disorder$.mp. or exp mental disease/
14. exp mental health/ or exp mental stress/ or psychological health.mp.
15. stress, psychological.mp.
16. psychological stress.mp.
17. psychological distress.mp. or exp distress syndrome/
18. adjustment disorder.mp. or exp adjustment disorder/
19. maladjustment.mp. or exp MALADJUSTMENT/
20. low mood.mp.
21. mental distress.mp. or exp distress syndrome/
22. distress.mp.
23. coping.mp. or exp coping behavior/
24. exp ANXIETY NEUROSIS/ or neurosis.mp. or exp AFFECTIVE NEUROSIS/ or exp NEUROSIS/
25. (sub?threshold and (depression or anxiety or depressive disorder$ or anxiety disorder$)).mp. [mp=title, abstract, subject headings, heading word, drug trade name, original title, device manufacturer, drug manufacturer, device trade name, keyword]
26. depressive symptom$.mp.
27. cognition disorder.mp. or exp cognitive defect/
28. (panic adj disorder).mp. [mp=title, abstract, subject headings, heading word, drug trade name, original title, device manufacturer, drug manufacturer, device trade name, keyword]
29. neurotic disorder.mp.
30. geriatric depression.mp.
31. copd.mp. or exp chronic obstructive lung disease/
32. obstructive lung disease.mp.
33. COAD.mp.
34. chronic obstructive airflow disease.mp.
35. Pulmonary disease, chronic obstructive.mp.
36. (chronic adj obstructive adj pulmonary adj disease).mp. [mp=title, abstract, subject headings, heading word, drug trade name, original title, device manufacturer, drug manufacturer, device trade name, keyword]
37. exp behavior therapy/ or exp cognitive therapy/ or cbt.mp.
38. exp PSYCHOSOCIAL REHABILITATION/ or exp PSYCHOSOCIAL DEVELOPMENT/ or exp PSYCHOSOCIAL CARE/
39. ((psychosocial or psycho-social) adj (program$ or therap$ or change$ or outcome$ or variable$ or factor$ of change$ or treatment$ or rehabilitation)).tw.
40. ((psychoeducational or psycho-educational) adj (program$ or therap$ or change$ or outcome$ or variable$ or factor$ of change$ or treatment$ or rehabilitation)).tw.
41. (behavio$r$ adj therap$).tw.
42. (behavio$r$ adj modif$).tw.
43. autogenic training.mp. or exp autogenic training/
44. counselling.mp. or exp counseling/
45. exp motivation/ or motivational interview$.mp.
46. group psychotherapy.mp. or exp group therapy/
47. group therapy.mp.
48. relaxation technique$.mp. or exp relaxation training/
49. social support.mp. or exp social support/
50. support network.mp.
51. (lifestyle adj (change$ or management)).tw.
52. (self adj (management or care)).tw.
53. ((stress or anxiety or distress) adj management).tw.
54. (behavio?r$ adj (change$ or programe$)).tw.
55. ((structured or brief) adj (intervention or follow-up)).tw.
56. rehabilitation.mp. or exp PSYCHOSOCIAL REHABILITATION/ or exp REHABILITATION CARE/ or exp REHABILITATION/
57. psychotherapy.mp. or exp PSYCHOTHERAPY/
58. exp adaptive behavior/
59. exp MEDITATION/ or meditation.mp.
60. (cognitive adj behavio?r$ adj therap$).tw.
61. (cognitive adj behavio?r$ adj therap$).tw.
62. (psychological adj intervention$).tw.
63. (psychological adj treatment).tw.
64. clinical trial/
65. (randomized controlled trials as topic or clinical trials as topic or randomi?ed controlled trial).mp. [mp=title, abstract, subject headings, heading word, drug trade name, original title, device manufacturer, drug manufacturer, device trade name, keyword]
66. intervention study.mp. or exp intervention study/
67. controlled study/
68. experimental control$.mp.
69. control group$.mp. or exp control group/
70. control$ design.mp.
71. randomi?ed.mp.

**Medline search**

1. exp Depression/ or depression.mp.
2. depressive disorder.mp. or exp Depressive Disorder/
3. affective disorder$.mp.
4. dysthymic disorder.mp. or exp Dysthymic Disorder/
5. dysthymia.mp.
6. reactive depression.mp.
7. exp Depressive Disorder/
8. major depression.mp.
9. masked depression.mp.
10. moderate depression.mp.
11. recurrent depression.mp.
12. long term depression.mp.
13. minor depression.mp.
14. exp Depressive Disorder, Major/
15. mild depression.mp.
16. atypical depression.mp.
17. anxiety.mp. or exp Anxiety/
18. anxiety disorders.mp. or exp Anxiety Disorders/
19. anxiety management.mp.
20. (mixed anxiety and depression).mp. [mp=title, original title, abstract, name of substance word, subject heading word, unique identifier]
21. mental disorder$.mp. or exp Mental Disorders/
22. Mental Disorders/
23. mental disease.mp.
24. psychological health.mp.
25. psychological health.mp.
26. mental health.mp. or exp Mental Health/
27. psychological stress.mp. or exp Stress, Psychological/
28. psychological distress.mp.
29. adjustment disorder.mp. or exp Adjustment Disorders/
30. maladjustment.mp.
31. mental stress.mp.
32. low mood.mp.
33. health anxiety.mp.
34. psychiatric symptom$.mp.
35. mental distress.mp.
36. distress.mp.
37. coping.mp.
38. mood disorders.mp. or exp Mood Disorders/
39. anxiety neurosis.mp. [mp=title, original title, abstract, name of substance word, subject heading word, unique identifier]
40. neurosis.mp.
41. distress syndrome.mp.
42. exp Neurotic Disorders/
43. coping behaviour.mp.
44. (sub?threshold and (depression or anxiety or depressive disorder$ or anxiety disorder$)).mp. [mp=title, original title, abstract, name of substance word, subject heading word, unique identifier]
45. exp Cognition Disorders/
46. (panic adj disorder).tw.
47. health anxiety.mp.
48. depressive symptom$.mp.
49. geriatric depression.mp.
50. panic disorder$.mp. or exp Panic Disorder/
51. (emotional adj (well-being or wellbeing or health or distress or dysfunction)).mp.
52. psychological dysfunction.mp.
53. ((psychoeducational or psycho-educational) adj (program$ or therap$ or change$ or outcome$ or effect$ or treatment$ or variable$ or factor$)).mp.
54. ((psychosocial or psycho-social) adj (program$ or therap$ or change$ or outcome$ or variable$ or factor$ of change$ or treatment$ or rehabilitation)).mp.
55. (psychological adj (distress or stress or rehabilitation or variable$ of factor$ or adaptation$ or outcome$ or therap$ or change$ of effect$)).mp.
56. (behavio$r$ adj therap$).mp.
57. (behavio?$ adj modif$).mp.
58. psychopathology.mp.
59. autogenic training.mp.
60. counsel?ing.mp.
61. cognitive therapy.mp. or exp Cognitive Therapy/
62. ((health or patient) adj education).mp.
63. motivational interview$.mp.
64. motivational interviewing.mp.
65. group psychotherapy.mp. or exp Psychotherapy, Group/
66. relaxation technique$.mp.
67. telephone.mp. or exp Telephone/
68. (tele$ adj (health or counsel$ or therap$ or treatment$ or advice$ or support$)).mp.
69. group therapy.mp. or exp Psychotherapy, Group/
70. (lifestyle adj (change$ or management)).mp.
71. (self adj (management or care)).mp.
72. ((stress or anxiety or distress) adj management).mp.
73. (behavio?r$ adj (change$ or program$)).mp.
74. health behavio?r.mp.
75. social support.mp. or exp Social Support/
76. support network$.mp.
77. ((structured or brief) adj (intervention or follow-up)).mp.
78. (nurse-led or nurse delivered).mp.
79. (nurse-led or nurse delivered).mp.
80. case-management.mp.
81. ((psychosocial or psycho-social) adj nursing).mp.
82. liaison nur$.mp.
83. Pulmonary rehabilitation.mp.
84. Rehabilitation.mp. or exp Rehabilitation/
85. Psychotherapy.mp. or exp Psychotherapy/
86. Counseling.mp. or exp Counseling/
87. Relaxation Techniques.mp. or exp Relaxation Therapy/
88. exp Psychotherapy, Group/
89. exp Adaptation, Psychological/
90. exp Patient Education as Topic/
91. exp Cognitive Therapy/
92. exp Health Behavior/
93. exp Rehabilitation, Vocational/
94. exp Stress, Psychological/
95. Stress.mp.
96. Lifestyle.mp. or exp Life Style/
97. lifestyle intervention.mp.
98. Health education.mp. or exp Health Education/
99. exp Health Promotion/
100. exp Life Change Events/
101. exp Motivation/
102. exp Social Support/
103. Social environment.mp. or exp Social Environment/
104. Self-care.mp. or exp Self Care/
105. exp Meditation/
106. exp Anxiety/
107. Anxiety management.mp.
108. exp Psychopathology/
109. exp Autogenic Training/
110. exp Behavior Therapy/
111. CBT.mp.
112. cognitive behavio?r$ therap$.mp.
113. psychological intervention$.mp.
114. psychological treatment$.mp.
115. Clinical Trial.mp. or exp Clinical Trial/
116. exp Randomized Controlled Trials as Topic/ or exp Randomized Controlled Trial/ or Randomized.mp.
117. placebo.mp. or exp Placebo Effect/
118. dt.mp.
119. randomly.mp.
120. trial.mp.
121. groups.mp.
122. exp Clinical Trial/
123. randomi?ed controlled trial.mp.
124. controlled trial.mp.
125. controlled clinical trial.mp. or exp Controlled Clinical Trial/
126. exp Intervention Studies/
127. exp Program Evaluation/
128. cluster randomi?ed controlled trial$.mp. [mp=title, original title, abstract, name of substance word, subject heading word, unique identifier]
129. (controlled study or studies).mp. [mp=title, original title, abstract, name of substance word, subject heading word, unique identifier]
130. experimental control$.mp.
131. experimental control$.mp.
132. control$ design.mp.
133. control$ design.mp.
134. control group$.mp.
135. control group$.mp.
136. pulmonary disease.mp.
137. (chronic adj obstructive adj pulmonary adj disease).mp.
138. COPD.mp.
139. COAD.mp.
140. exp Pulmonary Disease, Chronic Obstructive/
141. Chronic Obstructive Pulmonary Disease.mp.
142. Chronic Obstructive Pulmonary Disease.tw.
143. Obstructive lung disease.mp. or exp Lung Diseases, Obstructive/
144. Chronic airway obstruction.mp.
145. Airflow limitation.mp.
146. Chronic obstructive airway disease.mp.

**Psycinfo**

1. cognitive therapy/
2. cognitive behavior therapy/
3. cbt.mp.
4. psychotherapy.mp. or exp Psychotherapy/
5. group psychotherapy/
6. group psychotherapy/
7. intervention/
8. psychotherapeutic techniques/
9. psychotherapy/
10. psychosocial interventions.mp.
11. psychosocial readjustment/
12. counseling/
13. counseling psychology/
14. therapeutic processes/
15. educational counseling/
16. counselors/
17. psychotherapeutic counseling/
18. counselling.mp.
19. cardiac counselling.mp.
20. group therapy.mp.
21. anxiety management/
22. nurse-led.mp.
23. nurse-delivered.mp.
24. psychosocial nursing.mp.
25. chronic obstructive pulmonary disease/
26. copd.mp.
27. recurrent depression/
28. depression.mp.
29. depressive disorder.mp.
30. moderate depression.mp.
31. minor depression.mp.
32. mild depression.mp.
33. anxiety/
34. anxiety disorders/
35. exp Anxiety Management/
36. health anxiety.mp.
37. exp Panic Disorder/
38. rct.mp.
39. randomized controlled trial.mp.
40. exp Clinical Trials/

**Cinahl search**

1. (MH "Depression+")
2. depressive disorder
3. (MH "Affective Disorders+") OR (MH "Affective Disorders, Psychotic+")
4. (MH "Dysthymic Disorder")
5. dysthymia OR (MH "Dysthymic Disorder")
6. (MH "Depression, Reactive")
7. major depression
8. masked depression
9. moderate depression
10. recurrent depression
11. long term depression
12. minor depression
13. mild depression
14. atypical depression
15. (MH "Anxiety+") OR anxiety OR (MH "Anxiety Disorders+") OR (MH "Social Anxiety Disorders")
16. anxiety management
17. mixed anxiety and depression
18. (MH "Mental Disorders+") OR mental disorders OR (MH "Mental Disorders, Chronic")
19. mental disease
20. psychological health
21. (MH "Mental Health") OR mental health OR (MH "Research, Mental Health")
22. (MH "Stress, Psychological+") OR psychological stress
23. psychological distress OR (MH "Psychological Well-Being")
24. (MH "Adjustment Disorders+") OR adjustment disorder
25. maladjustment
26. mental stress
27. low mood
28. health anxiety
29. (MH "Affective Symptoms+") OR (MH "Behavioral Symptoms+") OR psychiatric symptoms
30. mental distress
31. distress
32. (MH "Coping+") OR coping
33. mood disorders
34. anxiety neurosis
35. neurosis
36. distress syndrome
37. (MH "Neurotic Disorders+") OR neurotic disorders
38. coping behaviour
39. (MH "Cognition Disorders+") OR cognition disorders
40. (MH "Panic Disorder")
41. health anxiety
42. depressive symptoms
43. geriatric depression
44. psychological dysfunction
45. (MH "Psychoeducation") OR psychoeducational
46. psychotherapautic therapy
47. psychotherapautic therapy
48. psycho therapy
49. psycho social
50. treatments
51. (MH "Rehabilitation+") OR rehabilitation
52. (MH "Behavior Therapy+") OR (MH "Alternative Therapies+") OR behaviour therapy
53. (MH "Behavior Modification+") OR behaviour modification
54. (MH "Psychopathology")
55. (MH "Autogenic Training (Iowa NIC)")
56. (MH "Counseling+") OR counselling
57. (MH "Cognitive Therapy")
58. (MH "Motivational Interviewing") OR motivational interview
59. (MH "Psychotherapy, Group+") OR (MH "Psychotherapy, Brief")
60. (MH "Relaxation Techniques+")
61. (MH "Telephone")
62. group therapy
63. lifestyle management
64. (MH "Self Care+") OR self management
65. (MH "Behavioral Changes") OR (MH "Life Style Changes") OR behaviour change
66. (MH "Health Behavior+") OR health behaviour
67. (MH "Social Support (Iowa NOC)") OR social support
68. (MH "Support, Psychosocial+") OR support networks
69. (MH "Intervention Trials") OR structured intervention
70. brief intervention
71. nurse delivered
72. case management
73. psychosocial nursing
74. (MH "Nurse Liaison") OR liaison nurse
75. (MH "Rehabilitation, Pulmonary+") OR pulmonary rehabilitation
76. (MH "Rehabilitation+") OR rehabilitation
77. (MH "Psychotherapy+") OR (MH "Psychotherapy, Brief")
78. (MH "Adaptation, Psychological+")
79. (MH "Patient Education+")
80. (MH "Rehabilitation, Vocational+")
81. (MH "Stress+")
82. (MH "Life Style+")
83. lifestyle intervention
84. (MH "Health Education+")
85. (MH "Health Promotion+")
86. (MH "Life Change Events+")
87. motivation
88. (MH "Social Environment+")
89. (MH "Meditation")
90. CBT
91. cognitive behaviour therapy
92. psychological intervention
93. psychological treatment
94. (MH "Clinical Trials+") OR randomized controlled trials
95. controlled clinical trial
96. rct
97. randomised controlled trials
98. (MH "Pulmonary Disease, Chronic Obstructive+") OR pulmonary disease
99. COPD
100. COAD
101. (MH "Lung Diseases, Obstructive+")
102. chronic airway obstruction
103. airflow limitation
104. chronic obstructive airway disease

**Web of Science search**

1. Depression
2. depressive disorder
3. anxiety
4. mental disorders
5. mental disease
6. stress
7. low mood
8. psychiatric symptoms
9. distress
10. mood disorders
11. cognitive disorders
12. panic disorder
13. depressive symptoms
14. emotional well being
15. psychological dysfunction
16. Psychoeducation
17. therapy
18. treatment
19. rehabilitation
20. training
21. counselling
22. counselling
23. therapy
24. education
25. motivational interview
26. management
27. behaviour change
28. behavior
29. intervention
30. psychotherapy
31. therapy
32. health
33. vocational rehabilitation
34. lifestyle intervention
35. self care
36. meditation
37. CBT
38. cognitive behavior therapy
39. behaviour
40. Randomized controlled trials
41. randomized controlled trial
42. controlled clinical trial
43. pulmonary disease
44. chronic obstructive pulmonary disease
45. copd
46. coad
47. obstructive lung disease
48. chronic airway obstruction
49. airflow limitation
50. chronic obstructive airway disease

**Scopus search**

1. Depression
2. depressive disorder
3. anxiety
4. mental disorders
5. mental disease
6. stress
7. low mood
8. psychiatric symptoms
9. mood disorders
10. cognitive disorders
11. panic disorder
12. depressive symptoms
13. psychological dysfunction
14. Psychoeducation
15. therapy
16. treatment
17. rehabilitation
18. training
19. counselling
20. counselling
21. education
22. motivational
23. management
24. behavior
25. intervention
26. psychotherapy
27. self care
28. meditation
29. CBT
30. cognitive
31. behaviour
32. randomized controlled trials
33. randomized controlled trial
34. controlled clinical trial
35. chronic obstructive pulmonary disease
36. copd
37. coad
38. chronic airway obstruction
39. airflow limitation
40. chronic obstructive airway disease
